# Case of Sclerosing Angiomatoid Nodular Transformation (SANT) in a G6PD Patient: Discussion of Histological Differential Diagnosis

**DOI:** 10.1155/crip/4676661

**Published:** 2026-11-08

**Authors:** Laura Ling Ying Tan, Wei Qiang Leow

**Affiliations:** ^1^ Department of Anatomical Pathology Division of Pathology Singapore General Hospital Singapore sgh.com.sg

**Keywords:** case report, sclerosing angiomatoid nodular transformation, spleen

## Abstract

Sclerosing angiomatoid nodular transformation (SANT) is a benign entity of the spleen composed of numerous angiomatoid nodules separated by fibrotic and sclerotic stroma. It was first described in 2004, with less than 300 published cases. Radiological findings are not specific and histological examination is needed for the definitive diagnosis of SANT. To further highlight and elucidate the features of this recently described tumour, we herein present a case of this entity and discuss the histological differential diagnosis. The patient is a 51‐year‐old Chinese male who was incidentally found to have a lesion in the spleen while undergoing surveillance hepatobiliary ultrasound for chronic hepatitis B. CT scan of the abdomen and pelvis showed a 1.8 cm large ill‐defined splenic soft tissue nodule. Laparoscopic splenectomy was performed. On microscopic examination, the lesion was well‐circumscribed and composed of numerous round to convoluted and coalescing angiomatoid nodules with intervening fibrosclerotic stroma. Immunohistochemical staining with CD31, CD34 and CD8 highlighted three distinct vascular populations within these angiomatoid nodules. The disease pathogenesis of SANT is unknown, with some proposing that it might represent a reactive, nonneoplastic lesion secondary to chronic inflammation or vascular insult. Our patient has a history of G6PD deficiency and it is possible that a subclinical episode of mild haemolytic anaemia may cause hemodynamic disturbances in the spleen leading to formation of SANT. Although SANT has a pathognomonic histological appearance, there are many benign and malignant entities that may mimic it histologically such as inflammatory myofibroblastic tumour, IgG4‐related disease and EBV‐positive inflammatory follicular dendritic cell sarcoma. This case highlights the histological diagnostic complexity of SANT, and the histological differentials that need to be considered in the workup of such a splenic lesion so as to avoid misdiagnosis and allow for appropriate subsequent patient management.

## 1. Introduction

Sclerosing angiomatoid nodular transformation (SANT) is a benign entity of the spleen composed of numerous angiomatoid nodules, consisting of three different types of blood vessels, which are separated by fibrotic and sclerotic stroma. It was first described in 2004 [1], with less than 300 published cases [2]. SANT has been reported across a wide age range but more commonly affects those in the fifth decade of life and has a female predominance. It is often asymptomatic with most cases incidentally discovered on radiological investigations. On contrast‐enhanced computed tomography (CT) or ultrasound (US), SANT is a well‐defined solid lesion that shows radially arranged enhancing septa, creating a spoke‐wheel pattern of nodular enhancement [3]. However, these imaging findings are not specific and the preoperative diagnosis remains challenging, with radiological differential diagnosis including haemangiomas, hamartomas or angiosarcomas [4].

As such, histological examination is needed for the definitive diagnosis of SANT, and to exclude other benign or neoplastic entities that occur in the spleen. To further highlight and elucidate the features of this recently described tumour, we herein present a case of this entity and discuss the histological differential diagnosis that needs to be considered.

## 2. Case presentation

The patient is a 51‐year‐old Chinese male, with multiple drug allergies, who has a past medical history of G6PD deficiency and iron deficiency anaemia and associated melena, for which esophagogastroduodenoscopy and colonoscopy performed was normal. He is also a chronic hepatitis B carrier for which he undergoes regular surveillance hepatobiliary US scans to monitor for liver cirrhosis.

During his most recent surveillance hepatobiliary US, an incidental non‐specific hypoechoic focus was noted in the spleen. As such, a CT scan of his abdomen and pelvis was performed, which showed a 1.8 cm large ill‐defined splenic soft tissue nodule. This splenic nodule was noted retrospectively on prior CT scan performed 12 months earlier (which was completed as part of his workup for iron deficiency anaemia and associated melena), where the lesion was smaller at 0.7 cm. Given the size increase over a year, the radiological report suggested exclusion of a malignant process.

He subsequently underwent a laparoscopic splenectomy. The intraoperative findings were that of a 1.8 cm splenic lesion, with no other gross lesions seen. The patient′s postoperative recovery was uneventful and he was discharged on postoperative Day 4.

The splenectomy specimen was sent to our laboratory for histological analysis. On macroscopic examination, the specimen consisted of a spleen weighing 83 g and measuring 10.8 cm × 6.5 cm × 5.2 cm. The visceral surface of the spleen, near the splenic notch, showed a firm exophytic nodular lesion. Cut sections revealed a firm, fairly well‐circumscribed, tan to reddish nodular lesion with a central greyish stellate fibrous area, which measured 2.7 cm × 2.6 cm × 2.3 cm (Figure 1). The rest of the splenic parenchyma appeared unremarkable. The entire splenic lesion was submitted for histological evaluation.

**Figure 1:**
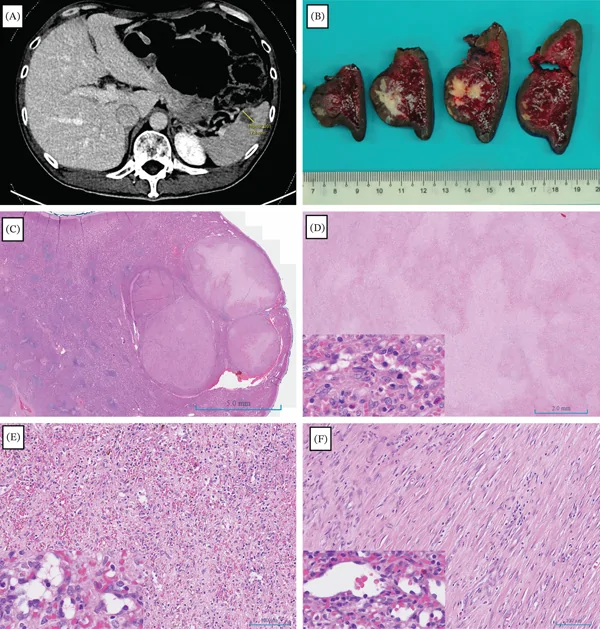
(A) Computed tomography (CT) scan of the abdomen and pelvis showed a 1.8 cm large ill‐defined splenic soft tissue nodule. (B) Cut sections of the splenectomy specimen revealed a fairly well‐circumscribed, tan to reddish nodular lesion with a central greyish stellate fibrous area. Photomicrographs of haematoxylin and eosin stained section of tumour. (C) On low power examination, a well‐circumscribed lesion was seen with normal background splenic parenchyma (2.5x magnification). (D) Angiomatoid nodules were seen in a sclerotic background, surrounded by concentric rings of collagen fibres (5x magnification). The inset highlights the vascular channels at higher power. (E) The angiomatoid nodule contained vascular channels reminiscent of splenic sinuses with surrounding extravasated red blood cells (100x magnification). The inset highlights the vascular channels at higher power. (F) The intervening fibrosclerotic stroma contained a mixture of inflammatory cells (100x magnification). The inset highlights the vascular channels at higher power.

On microscopic examination, the lesion was well‐circumscribed and nonencapsulated. It was composed of numerous round to convoluted and coalescing angiomatoid nodules with intervening fibrosclerotic stroma (Figure 1). These angiomatoid nodules contained a mixture of branching, dilated and slit‐like vessels which were lined by small bland uniform endothelial cells with no evidence of cytologic atypia or multilayering. There were numerous erythrocytes within and around these vessels. The fibrosclerotic stroma contained a mixture of inflammatory cells including lymphocytes, plasma cells, eosinophils and hemosiderin‐laden macrophages.

Immunohistochemical staining highlighted three distinct vascular populations within these angiomatoid nodules: capillaries expressing CD34 and CD31 but lacking CD8, sinusoidal channels that are CD8 and CD31 positive but CD34 negative and small venous structures that demonstrate CD31 positivity alone, without expression of CD34 or CD8 (Figure 2). CD8 also highlighted scattered T cells within the angiomatoid nodules. SMA highlighted myofibroblasts within the angiomatoid nodules. CD68 highlighted scattered macrophages within the angiomatoid nodules and fibrosclerotic stroma. These findings support the diagnosis of SANT of the spleen.

**Figure 2:**
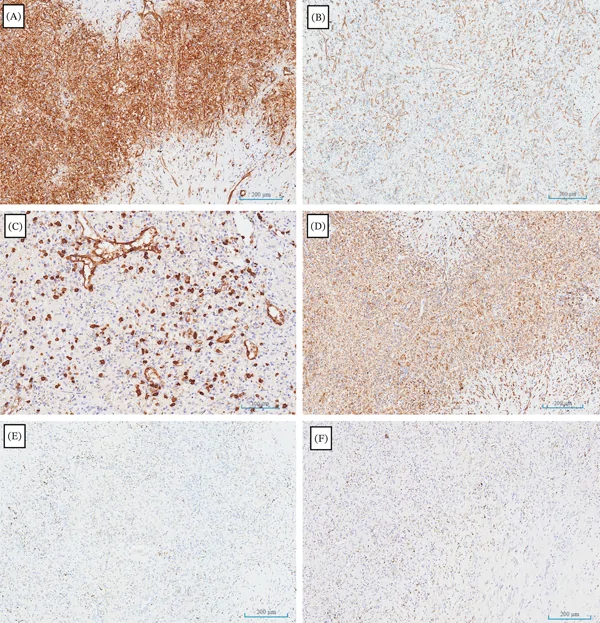
Photomicrographs of immunoperoxidase stained sections of tumour. (A) Staining with CD31 is positive in the capillaries, sinusoids and small veins in the angiomatoid nodules. (B) CD34 highlights capillaries in the angiomatoid nodules. (C) CD8 highlights sinusoids in the angiomatoid nodules. (D) CD68 highlights scattered macrophages within the angiomatoid nodules. (E) Staining with ALK was negative in the angiomatoid nodules. (F) In‐situ hybridisation for Epstein–Barr virus‐encoded RNA (EBER‐ISH) was negative in the angiomatoid nodules. (All photomicrographs were captured at 50x magnification except Figure 2(C) which was captured at 100x magnification.)

The lesion was negative for CD21 (providing no support for a follicular dendritic cell sarcoma [FDCS]), MNF116 (providing no support for a metastatic carcinoma), HHV8 (providing no support for Kaposi sarcoma), ALK (providing no support for inflammatory myofibroblastic tumour [IMT]) and SOX10 (providing no support for metastatic melanoma). The lesion was negative for EBER by in‐situ hybridisation (providing no support for EBV‐positive inflammatory FDCS). The background spleen was normal.

## 3. Discussion

Despite the numerous case reports of SANT in the literature, its disease pathogenesis and aetiology remain unclear. Previous studies have postulated that SANT might represent a reactive, nonneoplastic lesion secondary to chronic inflammation or vascular insult in which injury to small splenic vessels triggers the creation of haemangioma‐like vascular nodules, which subsequently undergo fibrous obliteration [5]. This proposed benign reactive nature of SANT is supported by its indolent clinical course, with no reported recurrence or metastasis in the literature [4]. Additionally, the vascular expression pattern in SANT (consisting of capillaries, sinusoids and small veins with characteristic staining patterns using a combination of CD31, CD34 and CD8) recapitulates the normal composition of splenic red pulp, supporting a nonneoplastic origin.

Although many currently regard SANT as benign, other studies have found that SANT contains exon deletions in the CTNNB1 gene, which encodes beta‐catenin [6], and may lead to the activation of the WNT/beta‐catenin signalling pathway [6]. SANT has also been found in association with visceral and haematolymphoid malignancies [1].

In our case, the patient had a history of G6PD deficiency and multiple drug allergies. Although he has had no reported history of acute haemolytic anaemia, it is possible that he could have experienced mild subclinical episodes of latent haemolysis secondary to triggers like infection or stress, which has been reported to occur in G6PD deficiency [7]. As the spleen is responsible for removing damaged red blood cells in haemolytic anaemia, it is possible that in an episode of mild haemolytic anaemia in a G6PD patient, the damaged red blood cells filtering through the splenic sinusoids may cause hemodynamic disturbances, leading to a regenerative response and the subsequent formation of SANT.

Although SANT has a pathognomonic histological appearance, there are many benign and malignant entities that may mimic it histologically. SANT may mimic other benign vascular lesions such as splenic hamartoma, splenic haemangioma and littoral cell angioma. Microscopically, littoral cell angioma shows anastomosing blood‐filled spaces with papillary fronds and no nuclear atypia, lacking the striking angiomatoid nodules seen in SANT. Littoral cell angioma shows a dual immunophenotype with co‐expression of endothelial markers like CD31 and ERG together with histiocytic markers (CD68), while being negative for CD34 [8]. On the other hand, the angiomatoid nodules in SANT shows a classic immunophenotype with capillaries that are CD34+/CD8‐/CD31+, sinusoids that are CD34‐/CD8+/CD31+ and small veins that are CD34‐/CD8‐/CD31+. Although littoral cell angioma and SANT are both benign lesions, littoral cell angioma has been reported to have recurrence or metastasis years after initial resection of the splenic lesion [8]. As such, distinguishing a littoral cell angioma from a SANT may affect the degree of future surveillance for the patient. Splenic haemangioma consists of disorganised blood‐filled vascular channels, lacking the multiple distinct angiomatoid nodules and concentric fibrous bands seen in SANT. Splenic haemangioma also differs from SANT immunohistochemically, consisting of only a single vessel type which is CD34+/CD8‐/CD31+.

Although splenic hamartoma similarly contains haphazardly arranged small slit‐like vascular spaces, it lacks the discrete angiomatoid nodules of SANT. The lining cells of the vascular spaces in splenic hamartoma are typically positive for CD8, CD31, CD34 and vimentin [9].

Additionally, the storiform fibrosis seen in IgG4‐related disease may mimic the concentric rings of collagen fibres surrounding the angiomatoid nodules in SANT. However, SANT lacks the other classic findings of IgG4‐related disease such as a prominent lymphoplasmacytic infiltrate or obliterative phlebitis. Immunohistochemical staining with IgG4 and IgG can be considered in cases of SANT with prominent fibrosclerotic stroma or with a prominent plasma cell component in the stroma. However, staining with IgG4 should be used with caution as a previous study of 22 cases of SANT found that 11% had increased IgG4+ cells but no patients had evidence of IgG4‐related disease. Although IgG4‐related is a systemic disease with often multiple affected organs and may require treatment with corticosteroids, surgical resection is curative for SANT.

The inflamed fibrosclerotic stroma and presence of myofibroblasts seen in SANT may mimic IMT, which has rarely been reported to occur in the spleen [10]. Some cases of IMT have a hypocellular fibrous pattern, which features hyalinized collagenous stroma with a lower cellularity of spindle cells, mimicking SANT. Unlike SANT, the myofibroblasts in IMT may have a ganglion‐like appearance with large rounded nuclei and prominent nucleoli. Approximately 50% and 10% of IMTs harbour *ALK* and *ROS1* gene rearrangements, respectively [11]. ROS1 IHC has limited utility as a biomarker for *ROS1* rearrangement. As such, to rule out IMT, molecular techniques such as FISH or RNA fusion testing should be used.

Various malignant conditions also need to be considered in the histological workup of SANT. EBV‐positive inflammatory FDCS, which occurs mainly in Asians and almost exclusively in the liver and spleen, consists of singly dispersed neoplastic spindle cells or loose whorled fascicles of spindle cells in a lymphoplasmacytic infiltrate [12], which may mimic the fibrosclerotic stroma of SANT. EBV‐positive inflammatory FDCS may also contain ectatic vessels, mimicking a vascular splenic lesion. As such, follicular dendritic cell markers like CD21 and EBER in‐situ hybridisation, which are consistently positive in EBV‐positive inflammatory FDCS, should be done to rule out this condition.

Kaposi sarcoma and SANT can both show a proliferation of small slit‐like vessels with extravasated erythrocytes and hemosiderin deposition. However unlike SANT, the endothelial cells in Kaposi sarcoma have minimal to moderate atypia, occasional hyaline globules and there may be surrounding intersecting fascicles of spindle cells. HHV8 can also be done to rule out Kaposi sarcoma. SANT lacks the multilayering of endothelial cells, nuclear pleomorphism, high mitotic rate, necrosis and poorly‐demarcated borders which is seen in angiosarcoma.

In conclusion, this case highlights the histological diagnostic complexity of SANT, and the morphological and immunohistochemical features that can help us differentiate SANT from its histological mimics. These histological differentials need to be considered in the workup of a splenic lesion showing either vascular spaces or an inflamed fibrosclerotic stroma so as to avoid misdiagnosis and allow for appropriate subsequent patient management.

## Funding

This study was supported by the SingHealth Duke‐NUS Pathology Academic Clinical Programme.

## Disclosure

During the preparation of this work, the authors did not use any artificial intelligence tools or technologies.

## Consent

The requirement for written informed consent was waived in accordance with the ethical guidelines of our institution.

## Conflicts of Interest

The authors declare no conflicts of interest.

## Supporting information


**Supporting Information** Additional supporting information can be found online in the Supporting Information section. Data S1: CARE checklist for this case report.

## Data Availability

The data that support the findings of this study are available from the corresponding author upon reasonable request.
