# Supplementary material for: Case of Sclerosing Angiomatoid Nodular Transformation (SANT) in a G6PD Patient: Discussion of Histological Differential Diagnosis
Source: Case Rep Pathol. 2026 Nov 8;2026:4676661. doi: 10.1155/crip/4676661 (PMC13745986; doi:10.1155/crip/4676661)
Supplement: Supplementary file 1 [file CRIP-2026-4676661-s001.docx]

**Supplementary Information 1. CARE checklist for “Case of Sclerosing angiomatoid nodular transformation (SANT) in a G6PD patient: discussion of histological differential diagnosis”**

| **Topic** | **Item No** | **Checklist item description** | **Reported on Page Number/Line Number** |
| --- | --- | --- | --- |
| Title | 1 | The diagnosis or intervention of primary focus followed by the words “case report” | Page 1/ Line 3 |
| Key Words | 2 | 2 to 5 key words that identify diagnoses or interventions in this case report, including "case report" | Page 1/ Line 11 |
| Abstract  (Structured summary) | 3a | Background: state what is known and unknown; why the case report is unique and what it adds to existing literature. | Page 2/ Line 27-32 |
|  | 3b | Case Description: describe the patient’s demographic details, main symptoms, history, important clinical findings, the main diagnosis, interventions, outcomes and follow-ups. | Page 2/ Line 33-36 |
|  | 3c | Conclusions: summarize the main take-away lesson, clinical impact and potential implications. | Page 2/ Line 45-50 |
| Introduction | 4 | One or two paragraphs summarizing why this case is unique **(may include references)** | Page 3/ Line 60-73 |
| Patient Information | 5a | De-identified patient specific information | Page 3/ Line 75-79 |
|  | 5b | Primary concerns and symptoms of the patient | Page 3/ Line 80-82 |
|  | 5c | Medical, family, and psycho-social history including relevant genetic information | Page 3/ Line 75-76 |
|  | 5d | Relevant past interventions with outcomes | N/A |
| Clinical Findings | 6 | Describe significant physical examination (PE) and important clinical findings | N/A |
| Timeline | 7 | Historical and current information from this episode of care organized as a timeline | N/A |
| Diagnostic Assessment | 8a | Diagnostic testing (such as PE, laboratory testing, imaging, surveys). | Page 3/ Line 80-86 |
|  | 8b | Diagnostic challenges (such as access to testing, financial, or cultural) | N/A |
|  | 8c | Diagnosis (including other diagnoses considered) | Page 5-6/ Line 140-189 |
|  | 8d | Prognosis (such as staging in oncology) where applicable | N/A |
| Therapeutic Intervention | 9a | Types of therapeutic intervention (such as pharmacologic, surgical, preventive, self-care) | N/A |
|  | 9b | Administration of therapeutic intervention (such as dosage, strength, duration) | N/A |
|  | 9c | Changes in therapeutic intervention (with rationale) | N/A |

| Follow-up and Outcomes | 10a | Clinician and patient-assessed outcomes (if available) | Page 3/ Line 88-89 |
| --- | --- | --- | --- |
|  | 10b | Important follow-up diagnostic and other test results | N/A |
|  | 10c | Intervention adherence and tolerability (How was this assessed?) | N/A |
|  | 10d | Adverse and unanticipated events | N/A |
| Discussion | 11a | A scientific discussion of the strengths AND limitations associated with this case report | N/A |
|  | 11b | Discussion of the relevant medical literature **with references** | Page 4-5/ Line 119-139 |
|  | 11c | The scientific rationale for any conclusions (including assessment of possible causes) | Page 4-5/ Line 119-138 |
|  | 11d | The primary “take-away” lessons of this case report (without references) in a one paragraph conclusion | Page 6/ Line 190-194 |
| Patient Perspective | 12 | The patient should share their perspective in one to two paragraphs on the treatment(s) they received | See below. |
| Informed Consent | 13 | Did the patient give informed consent? Please provide if requested | See below. |

Regarding CARE checklist points 12 & 13:

Our publication has been prepared in accordance with our institution’s “Requirements for Preparing a Case Report for Publication”, which states that a case report involving one or two patients is not defined as research by the centralized institutional review board (CIRB) and does not require CIRB review as it does not involve the formulation of a research hypothesis that is subsequently investigated prospectively and systematically for publication or presentation. Written informed consent would not be required if direct identifiers are not included in the submission and publication of the report. Written informed consent is required if the patient(s) in the case report may be identified from the case report, even if it does not have any of the direct identifiers. This may occur in (but not be limited to) the following situations: The disease is so rare that patient(s) with the disease may be identified easily. In this case, although rare, sclerosing angiomatoid nodular transformation of the spleen has been previously described in the literature and the patient would not be identified easily from the case report.
